# Phosphoproteomic Profiling of Multiple Myeloma Based on Ex Vivo Drug Sensitivity Resistance Testing Identifies Phosphorylation Signatures Associated with Drug Response

**DOI:** 10.3390/biom16020323

**Published:** 2026-02-19

**Authors:** Katie Dunphy, Ellen Purcell, Caroline A. Heckman, Paul Dowling, Despina Bazou, Peter O’Gorman

**Affiliations:** 1 Department of Biology, Maynooth University, W23 V5XH Kildare, Ireland; katie.dunphy.2015@mumail.ie (K.D.); ellen.purcell.2025@mumail.ie (E.P.); paul.dowling@mu.ie (P.D.); 2 Kathleen Lonsdale Institute for Human Health Research, Maynooth University, W23 V5XH Kildare, Ireland; 3 Institute for Molecular Medicine Finland-FIMM, HiLIFE, University of Helsinki, 00290 Helsinki, Finland; caroline.heckman@helsinki.fi; 4 School of Biomolecular and Biomedical Sciences, University College Dublin, D04 V1W8 Dublin, Ireland; 5 Department of Haematology, Mater Misericordiae University Hospital, D07 R2WY Dublin, Ireland

**Keywords:** drug resistance, filamin, mass spectrometry, multiple myeloma, phosphorylation, protein kinase A proteomics, resistance, sensitivity

## Abstract

Multiple myeloma (MM) is characterised by the clonal expansion of plasma cells in the bone marrow followed by end-organ damage. Despite a significant increase in the five-year survival rate in recent years, MM is still considered an incurable disease as patients will repeatedly relapse and develop resistance to standard-of-care therapies. A central theme for the personalization of MM therapy is understanding the biological mechanisms of drug resistance and identifying clinically relevant biomarkers of therapeutic response. Highly effective protocols for the enrichment of phosphorylated peptides followed by high-resolution mass spectrometry makes possible the quantitation of thousands of site-specific phosphorylation events, principally on serine, threonine or tyrosine residues. In this study, phosphoproteomic analysis of 20 MM patient cell lysates was performed, stratified based on their ex vivo drug response profiles to Bortezomib and Lenalidomide, two of the most foundational therapeutic agents in the management of MM. In this study, patients who are highly sensitive to these drugs show increased phosphorylation of proteins concerned with translation and RNA processing including the spliceosome, RNA transport and RNA binding pathways, while highly resistant patients demonstrated an increased phosphorylation of proteins involved with tight junctions, the Rap1 signalling pathway and the phosphatidylinositol signalling system. This study has established a phosphoproteomic dataset displaying unique phosphorylation signatures associated with drug sensitivity in MM patient plasma cells. The identification of phosphorylation signatures associated with drug resistance provides the foundation for further exploration of these mechanisms and associated signalling pathways to further characterise drug resistance mechanisms in MM and identify promising biomarkers of therapeutic response and targets for drug re-sensitization in MM.

## 1. Introduction

Clinical proteomics is crucial for modern medicine, enabling early disease detection, precise molecular diagnosis, and personalised therapy by analysing protein expression patterns in bodily fluids and tissues. Using advanced techniques like mass spectrometry and bioinformatics, clinical proteomics aims to identify diagnostic biomarkers, monitor treatment efficacy, and uncover drug resistance mechanisms. Phosphoproteomics, a key branch of proteomics, focuses on studying protein phosphorylation at a global scale to understand its role in biological processes. Protein phosphorylation is one of the most extensively investigated post-translational modifications (PTMs) due to the key role it plays in almost all cellular processes, particularly in signal transmission. The majority of phosphorylation events occur on serine residues (~90%), followed by threonine residues (~9%), and a small percentage on tyrosine residues (<1%). Phosphorylation has also previously been discovered to occur on histidine, arginine, lysine, and cysteine residues, although such events are less documented [1]. The mechanism of amino acid phosphorylation is reversible, whereby protein kinases catalyse and protein phosphatases reverse phosphorylation, maintaining homeostasis. Alterations in the activity or abundance of kinases and phosphatases can have a significant impact on the magnitude of protein phosphorylation, which can directly impact protein functionality through changes in protein localisation, protein complex formation, signal transduction, or other biological processes. In oncology research, phosphoproteomic analyses contribute to precision medicine through identification of phosphorylation sites and associated kinases/phosphatases that can potentially act as biomarkers and therapeutic targets. The improvements in phosphoproteomic approaches have led to important advances in researchers’ understanding of cancer signalling dynamics. Numerous studies have identified phosphorylation events as promising biomarkers of therapeutic response and prognosis [2,3]. Consequently, phosphorylation signatures associated with distinct cancer subtypes or mutational profiles can be seen as contributing to precision medicine [4,5]. Mass spectrometry (MS) is an influential analytical technique applied in phosphoproteomic studies, facilitating large-scale, high-resolution, quantitative profiling of phosphorylated proteins. Despite being the most comprehensively studied PTM, MS-based analysis of phosphorylation events remains complex, primarily due to substoichiometric phosphorylation, which concerns the inherent underrepresentation of phosphopeptides when compared to their unmodified peptide counterpart in complex peptide mixtures [6]. This substoichiometry can impede phosphopeptide identification; however, the use of phosphopeptide enrichment methods (both antibody- and chemistry-based) and highly sensitive, high-resolution mass spectrometers have improved the capability of phosphoproteomics studies to detect greater numbers of phosphopeptides. Western blotting (WB) is commonly used for the targeted analyses of phosphorylation sites; however, a significant limitation of WB is the limited availability of high-quality, highly specific antibodies against many phosphorylation sites. There is a relatively small number of phosphor-specific antibodies commercially available, compared to the thousands of phosphorylation sites in the proteome, making them a “limited” resource in the context of total phosphorylation mapping. In this study, a phosphoproteomic approached was utilised to screen patient MM plasma cell lysates to identify novel sites associated with therapeutic response [7]. MM, the second most frequent hematologic malignancy, is characterised by the expansion of monoclonal plasma cells in the bone marrow. Patients present with bone lesions, renal insufficiency, hypercalcemia, and bone marrow failure (CRAB criteria) [8]. The introduction of efficacious therapies including proteasome inhibitors and immunotherapies in the past decade has substantially extended the survival of MM patients; however, MM remains terminal and those patients with high-risk cytogenetic characteristics have a markedly poor outcome. This study focuses on identifying phosphoproteomic signatures of MM patients being treated with two standard FDA-approved MM therapeutics—Bortezomib, a proteasome inhibitor (PI) and Lenalidomide, an immunomodulatory drug (IMiD).

## 2. Materials and Methods

### 2.1. Patient Samples and Clinical Information

**Figure 1:**
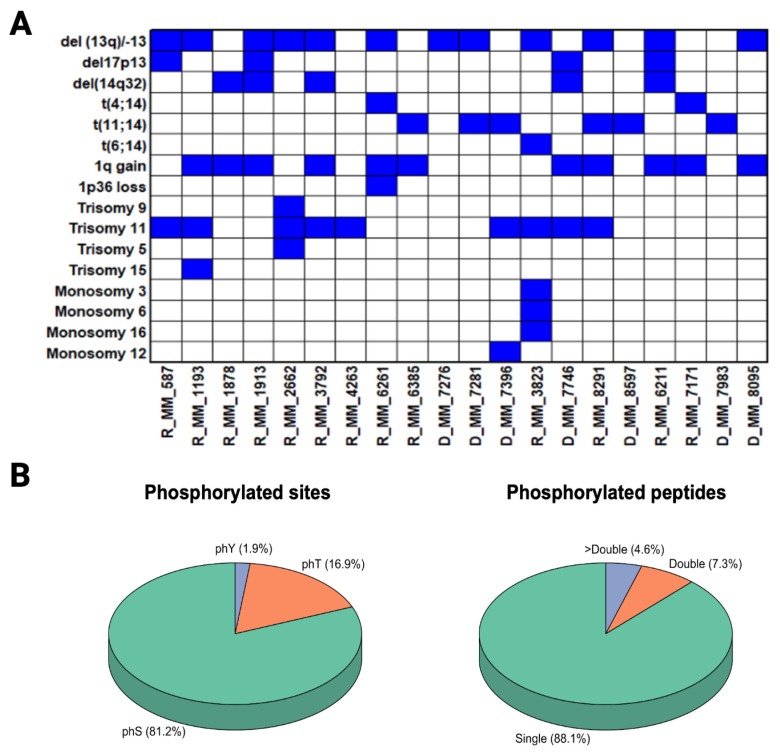
(**A**) Cytogenetic data associated with the patient cohort. Cytogenetic analysis was performed on the sampling date. We note that del (14q32) is quite rare, with an estimated incidence of ~1.4% of plasma cell malignancies [9,10], as opposed to 14q32 translocations, which are very frequent. In addition, there is little evidence of its prognostic significance or therapeutic implications. These samples and the associated clinical information were acquired from the Finnish Haematology Registry and Clinical Biobank (FHRB Biobank). Because del (14q32) is in fact uncommon, any racial difference, if it exists, has not been statistically demonstrated. Most racial disparity research in MM focuses on IGH translocations, hyperdiploidy, and mutational profiles, not on IGH deletions. The del (14q32) is frequent in the Finnish population; however, a larger patient is required to draw more solid results. (**B**) Visualisation of phosphorylated peptides and sites distribution. Distribution of serine (phS), threonine (phT), and tyrosine (phY) phosphorylation sites identified in this phosphopeptide analysis using enrichment and MS. Distribution of phosphopeptides with one, two, or greater than two phosphorylation sites identified in this study.

Patient samples were collected after informed consent with ethical approval from the contributing hospitals in compliance with the Declaration of Helsinki. Bone marrow aspirates were collected from 20 MM patients at various stages of disease and with varying response rates to therapy. Patient characteristics are outlined in Supplementary Table S1 along with information on their therapeutic regimes, as outlined in Supplementary Table S2. Cytogenetic information for each patient at the time of sampling was evaluated (Figure 1A).

### 2.2. Label-Based Mass Spectrometry Analysis of Primary CD138+ Myeloma Cells

The mononuclear cell fraction of bone marrow aspirates collected from 20 MM patients were subject to CD138+ plasma cell enrichment using the EasySep Human CD138 Positive Selection kit (StemCell Technologies, Grenoble, France) at the Institute for Molecular Medicine Finland (FIMM). Drug sensitivity scoring was performed at FIMM, as described previously [11,12,13]. In summary, CD138+ cells collected from the 20 MM patients were tested against a panel of compounds (>300 small molecule inhibitors) that were pre-plated on 384-well plates at five concentrations over tenfold dilutions covering a 10,000-fold concentration range (1–10,000 nM). CD138+ cells were added to the plates in conditioned medium prepared from the HS5 human bone marrow stromal cell line, widely used as a model bone marrow niche cell line. The plates were incubated in a humidified environment at 37 °C and 5% CO_2_ for 72 h, followed by measurement of cell viability using the CellTiter-Glo assay (Promega, Madison, WI, USA). Drug sensitivity data was calculated by comparing readouts between drug-treated and negative control (DMSO only)-treated cells. Drug sensitivity scores (DSSs) were calculated as previously described [14].

Isolated CD138+ plasma cells were lysed in RIPA buffer (25 mM Tris, pH 7–8; 150 mM NaCl; 0.1% SDS; 0.5% sodium deoxycholate and 1% NP-40) using a combination of vortexing and sonication. Protein quantitation was performed using the Pierce™ 660 nm protein assay (Thermo Fisher Scientific, Waltham, MA, USA), using the Ionic Detergent Compatibility Reagent (IDCR). Filter Aided Sample Preparation (FASP) was used for proteolytic digestion of sample lysates [15]. A total of 30 μg of protein from each sample lysate was digested using trypsin. For this mass spectrometry analysis, 50 mM HEPES, pH 8.5 was used as the digestion buffer instead of 50 mM Ammonium Bicarbonate (ABC) to ensure no interference with tandem mass tag (TMT) reagents. Following buffer exchange (with 50 mM HEPES, pH 8.5), overnight trypsin digestion was performed using a 1:30 enzyme-to-protein ratio using Sequencing Grade Modified Trypsin (V5111, Promega), in 50 mM HEPES, pH 8.5. Following overnight digestion, the filter units were transferred to new collection tubes and centrifuged at 14,000× *g* for 10 min. A total of 40 μL of 50 mM HEPES, pH 8.5 was added to all samples, followed by centrifugation at 14,000× *g* for 10 min to obtain ~80 μL of peptide eluate.

TMT labelling was performed immediately after protein digestion overnight. Two TMT10plex Isobaric Label Reagent Sets (Thermo Fisher Scientific Inc.) were used at a TMT label reagent-to-protein ratio of ~8:1. Following labelling, the pooled samples were partially dried, and acidified at a 1:7 ratio (1 part acidic sample buffer, 7 parts sample) using 2% TFA, 20% ACN. Sample clean-up to remove interfering contaminants was performed using Pierce C18 Spin Columns (Thermo Fisher Scientific Inc.), followed by sample drying using a speed vacuum. Dried labelled peptides were resuspended in 200 μL of Binding/Wash Buffer for phosphopeptide enrichment using the High-Select™ Fe-NTA Phosphopeptide Enrichment Kit (Thermo Fisher Scientific Inc.). Phosphopeptide enrichment produced both a phosphorylated and unphosphorylated fraction for each TMTplex of 10 pooled samples. Mass spectrometry analysis was performed using the Thermo UltiMate 3000 nano system directly coupled in-line with the Thermo Orbitrap Fusion Tribrid™ mass spectrometer (Waltham, MA, USA), using 6.4 µL of sample per injection.

Mass spectrometry analysis was performed using the Thermo UltiMate RSLC3000 nano system directly coupled in-line with the Thermo Orbitrap Fusion Tribrid™ mass spectrometer. For both the phospho-enriched and non-enriched samples, volumes equivalent to 1 µg of digested peptides (6.4 µL) were loaded onto the trapping column (PepMap100, C18, 300 μm × 5 mm; Thermo Fisher Scientific) for 3 min at a flow rate of 25 μL/min with 2% (*v*/*v*) ACN, 0.1% (*v*/*v*) TFA. Peptides were resolved on an analytical column (Easy-Spray C18 75 μm × 250 mm, 2 μm bead diameter column, Thermo Fisher Scientific, Waltham, MA, USA) using the following gradient: 98% solvent A (0.1% (*v*/*v*) formic acid in LC-MS grade water) to 35% solvent B (80% (*v*/*v*) ACN, 0.08% (*v*/*v*) formic acid in LC-MS grade water) over 120 min at a flow rate of 300 nL/min. The mass spectrometer was operated in data-dependent acquisition (DDA) mode with multi notch synchronous precursor selection MS3 scanning for TMTs. The scan sequence for the Orbitrap Fusion Tribrid began with the acquisition of MS1 spectra over 400–1400 *m*/*z* in the Orbitrap at a resolution of 120,000 at 200 *m*/*z*. Automatic gain control (AGC) was set to accumulate 4 × 10^5^ ions with a maximum injection time of 100 ms and 50 ms for the unenriched and phospho-enriched samples, respectively. MS2 analysis was performed in the ion trap using a top-speed approach with 3 s cycles. For the unenriched samples, an intensity threshold of 5000 was used and charge states 2+ to 7+ were included. For the phosphor-enriched samples, an intensity threshold of 10,000 was used and charge states 2+ to 6+ were included. Collision-induced dissociation (CID) fragmentation was applied and normalised collision energy was optimised at 35%. A dynamic exclusion of 50 s was applied with a mass tolerance of 10 ppm and the AGC target was set at 104. For MS3 analysis (synchronous precursor selection), precursors within the mass range 400 to 1200 *m*/*z* were selected, an isolation window of 2 *m*/*z* was used, and isobaric tag loss exclusion set for TMT. Selected precursors were fragmented by higher-energy collisional dissociation (HCD) (65% collisional energy) and detected using the Orbitrap over 100–500 *m*/*z* at a resolution of 60,000 at 200 *m*/*z*.

### 2.3. Drug Sensitivity and Resistance Testing (DSRT)

CD138+ plasma cells were enriched suing the EasySep Human CD138 Positive Selection Kit (StemCell Technologies, Grenoble, France) from the mononuclear cell fraction of BM aspirates following gradient separation (Ficoll-Paque PREMIUM; GE Healthcare, Little Chalfont, Buckinghamshire, UK). Drug sensitivity scoring (DSS) was performed based on methods previously described [11,12,13]. CD138+ plasma cells derived from MM patients were tested against 308 compounds at five concentrations over tenfold dilutions covering a 10,000-fold concentration range (1–10,000 nM). The drug panel included approved oncology drugs (*n* = 141) and investigational compounds (*n* = 167) targeting multiple signalling networks and molecular targets. In summary, 5 µL of cell culture medium containing RPMI 1640 medium supplemented with 10% foetal bovine serum, 2 mM L-glutamine, penicillin (100 U/mL), streptomycin (100 µg/mL) and 25% conditioned medium from the HS-5 human BM stromal cell line was added to the 384-well drug plates and shaken for 5 min to dissolve the compounds. CD138+ plasma cells were diluted in the cell culture medium and 20 µL of the cell suspension containing 5000 cells was transferred to each well using a MultiDrop Combi peristaltic dispenser (Thermo Scientific, Waltham, MA, USA). The plates were incubated in a humidified environment at 37 °C and 5% CO_2_. Cell viability was measured after 72 h using the CellTiter-Glo assay (Promega, Madison, WI, USA) with a PHERAstar microplate reader (BMG-Labtech, Offenburg, Germany) to measure luminescence. The mean viability of untreated cells at day three was 124 ± 10.40%. The data was normalised to negative (DMSO only) and positive control wells (containing 100 µM benzethonium chloride). Scoring and clustering of the DSRT data was performed as previously described by Pemovska and co-workers [8].

### 2.4. Data Analysis and Bioinformatic Analysis of Mass Spectrometry Results

To identify phosphorylation sites with a significant change in abundance levels, statistical analysis was performed using ANOVA or two-sided *t*-test with permutation-based FDR statistics. Statistically significant differentially abundant (SSDA) phosphorylation sites between the chemosensitivity groups were identified based on an FDR threshold < 0.05 (s0 = 0.1) and fold change > 1.5. For the analysis of individual drugs, phosphorylation sites were also identified based on an FDR threshold < 0.1 (s0 = 0.1) and fold change > 1.5. To identify the biological functions of SSDA phosphoproteins, the 2023 version SRPlot online platform (http://www.bioinformatics.com.cn/srplot, accessed on 1 November 2023) which is based on the “clusterProfiler” and “pathview” R packages, and g:Profiler gGOst (https://biit.cs.ut.ee/gprofiler/gost, accessed on 1 November 2023, version 2023) were used. The SRPlot online tool was used to visualise the gene ontology results. Kinase-substrate enrichment analysis (KSEA) was performed using the 2017 version KSEA App website (https://casecpb.shinyapps.io/ksea/, accessed on 1 November 2023) using PhosphoSitePlus and NetworKIN databases according to a NetworKIN cutoff of 1.5 and *p*-value cutoff of *p* < 0.05. Only kinases with a substrate count ≥ 3 are displayed on bar plots. To identify variant sequence motifs, the ±31 amino acid sequence windows of the significantly regulated phosphorylation sites were evaluated using the online software tool, MoMo (v5.5.3) (accessed 1 November 2023) [16]. A 31-residue motif width, 15 occurrences, and a *p*-value of <0.000001 were set as parameters for motif prediction.

## 3. Results

### 3.1. Stratification of Plasma Samples Based on Ex Vivo Drug Resistance Testing

Following ex vivo DSRT to a panel of >300 drugs, CD138+ myeloma cell samples were stratified into one of four groups: Group 1, very sensitive; Group 2, sensitive; Group 3, resistant; and Group 4, very resistant to the panel of >300 drugs employed in the DSRT platform as previously described [12,13] (Table 1). Cytogenetic information for each patient at the time of sampling was also available (Figure 1A).

### 3.2. Quantitative Phosphoproteomics of CD138+ Myeloma Cell Lysates

Quantitative phosphoproteomic mass spectrometry analysis identified a total of 1473 proteins and 2945 phosphorylation sites on 2232 phosphopeptides from 690 phosphoproteins. A stringent phosphorylation site localisation probability (>0.95) was used to ensure the inclusion of high-confidence phosphorylation sites for subsequent downstream statistical analyses. The phosphopeptide and phosphosite residue distribution was similar to previous studies [17,18,19] (Figure 1B). As expected, the majority of phosphorylation sites identified involved serine residues (81.2%).

### 3.3. Analysis of the Phosphoproteome of CD138+ Myeloma Cell Lysates Stratified into Four Chemosensitivity Groups

**Figure 2:**
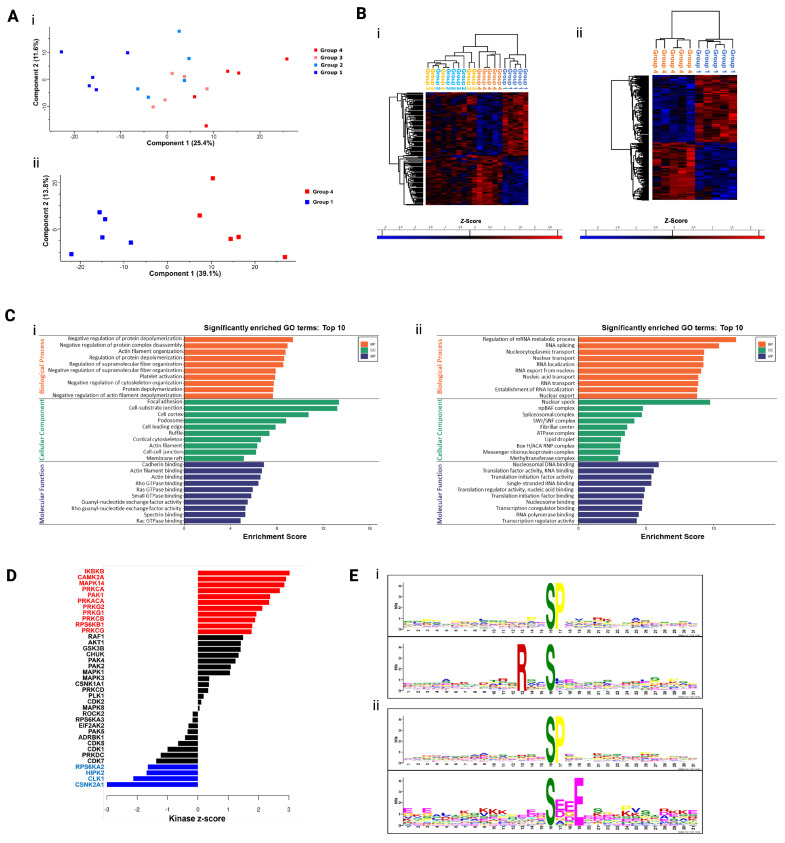
(**A**) Principal component analysis (PCA) of reporter ion intensity values from phosphopeptides identified in the four chemosensitivity groups. (**i**) PCA of phosphopeptides identified in Groups 1, 2, 3, and 4. (**ii**) PCA of phosphopeptides identified in Groups 1 and 4 highlighting the clear distinction between the very sensitive and very resistant groups. (**B**) Hierarchical clustering analysis of statistically significantly abundant (SSDA) phosphorylation sites. (**i**) Hierarchical clustering analysis z-scored normalised intensity values of the 152 SSDA phosphosites across the four chemosensitivity groups. (**ii**) Hierarchical clustering of z-scored normalised intensity values of the 217 SSDA phosphosites. (**C**,**i**) Gene ontology enrichment analysis of phosphoproteins found to be statistically significantly increased in Group 4. This graph highlights the top 10 most significantly enriched biological processes (orange), cellular components (green), and molecular functions (purple). (**ii**) Gene ontology enrichment analysis of phosphoproteins found to be statistically significantly increased in Group 1. The graph highlights the top 10 most significantly enriched biological processes (orange), cellular components (green), and molecular functions (purple). (**D**) Bioinformatic analysis of phosphorylation motifs and upstream kinases. Kinase-substrate enrichment analysis (KSEA) was performed to characterise kinase regulation based on drug resistance/sensitivity. Kinases with a *p*-value < 0.05 are highlighted as red and blue bars. Red bars indicate kinases predicted to be activated in drug-resistant (Group 4) myeloma cells, whereas blue bars indicate kinases predicted to be activated in drug-sensitive (Group 1) myeloma cells. (**E**,**i**) Significantly enriched phosphorylation motifs from the phosphopeptides significantly increase in abundance in Group 4 samples. (**ii**) Significantly enriched phosphorylation motifs from the phosphopeptides significantly increased in abundance in Group 1 samples.

Principal component analysis (PCA), a linear dimensionality reduction technique, was performed on the phosphopeptide intensity values, revealing a clear separation between Groups 1 and 4, highlighting a change in the phosphoproteome between these groups. Groups 2 and 3 demonstrated some overlap (Figure 2(Ai)), indicating a degree of similarity between the samples in these groups; however, samples from Groups 1 and 4 have notably different phosphoproteomic profiles (Figure 2(Aii)).

To evaluate the phosphoproteomic changes in MM patient plasma cells of the four chemosensitivity groups, we compared phosphopeptide abundance across the four groups using Analysis of Variance (ANOVA). Of the 2945 phosphorylation sites identified, 152 phosphorylation sites were SSDA between the four chemosensitivity groups (ANOVA q-value < 0.05). A Student’s *t*-test was also performed to identify the phosphorylation site when comparing the “very sensitive” and “very resistant” phenotypes. A total of 212 SSDA phosphorylation sites were identified (FDR q-value < 0.1, FC > 1.5). Hierarchical clustering, an unsupervised machine learning method that builds a tree-like hierarchy of clusters (dendrogram), was performed on z-scored intensity values to illustrate the changes in abundance of the SSDA phosphopeptides identified by ANOVA and Student’s *t*-test (Figure 2(Bi,Bii)). Two distinct clusters representing phosphorylation sites increased in abundance in Group 1 and Group 4 are clearly visible. SSDA phosphoproteins were separated into those increased in abundance in Group 4 and those increased in abundance in Group 1 and analysed further using gene ontology enrichment analysis. As shown in Figure 2(Ci,Cii), phosphoproteins increased in abundance in Group 4 are associated with cytoskeletal organisation, while those increased in Group 1 are associated with RNA binding and translation. Kinase-substrate enrichment analysis (KSEA) was used to predict potential kinase activity based on the phosphorylation levels of known substrates. This analysis showed significant enrichment of 11 kinases in drug-sensitive (Group 4) samples and significant enrichment of 4 kinases in drug-resistant (Group 1) samples (Figure 2D). Phosphorylation mechanisms are dependent on the action of protein kinases which recognise specific short (~5–15 amino acids)-sequence motifs surrounding the serine, threonine or tyrosine residues which are subsequently phosphorylated. A motif analysis was performed using MoMo and the motif-x algorithm to better understand the upstream processes of the phosphopeptides identified in this study (Figure 2(Ei,Eii)). MoMo discovers sequence motifs associated with distinct types of protein post-translational modifications (PTMs). One motif, namely S*P (* denotes phosphorylated residue), was significantly increased in both Group 4 and Group 1 phosphopeptides. The motif RxxS* was uniquely increased in phosphopeptides and increased in abundance in Group 4 samples, while the motif S*xxE was uniquely increased in phosphopeptides and increased in abundance in Group 1. The RxxS* motif is a phosphorylation site for protein kinase A (PKA) whose catalytic subunit (PRKACA) was significantly enriched in Group 4 samples from the KSEA [20]. Interestingly, the S*xxE motif is a consensus sequence motif of casein kinase (CK2) whose catalytic subunit (CSNK2A1) was significantly enriched in Group 1 samples from the KSEA.

### 3.4. Evaluation of MM Patient Response to Individual Drugs Based on Ex Vivo Drug Sensitivity Scores

**Figure 3:**
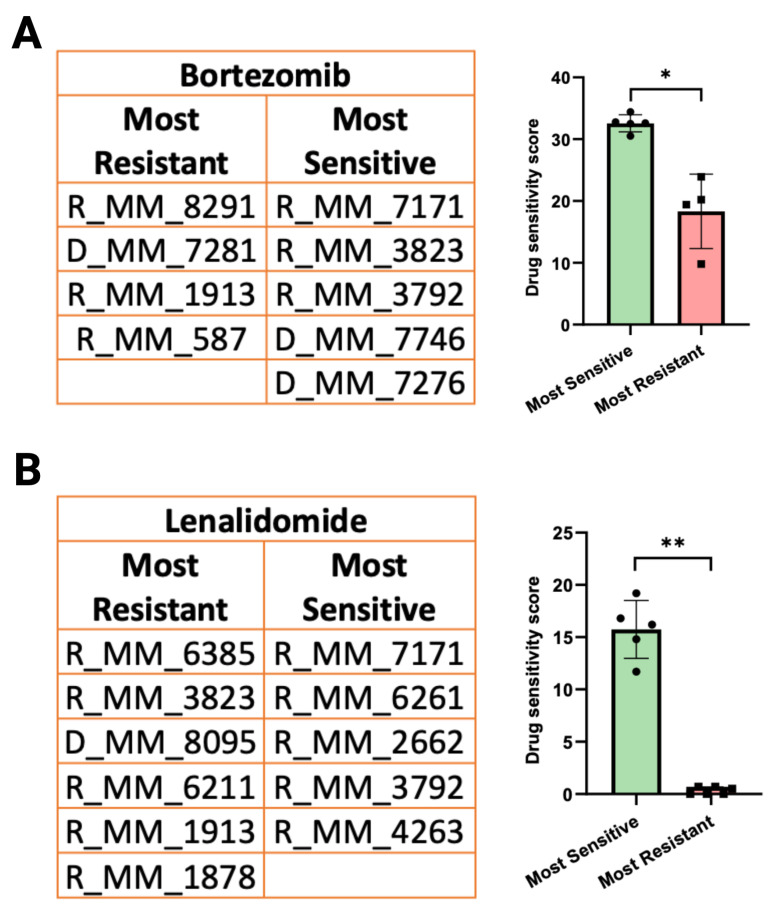
(**A**,**B**) Sample stratification into “most resistant” and “most sensitive” groups for Bortezomib and Lenalidomide. Samples were stratified into “most resistant” and “most sensitive” groups based on DSS values. The drug sensitivity scores in “most sensitive” and “most resistant” groups associated with each individual drug is also shown. Normality was determined using the Shapiro–Wilk test. Statistical significance was evaluated by an unpaired *t*-test with Welch correction. Significance is marked as follows: *p* ≤ 0.05 “*” and *p* ≤ 0.01 “**”.

To identify phosphoproteomic changes within MM patient cells that are associated with response to Bortezomib and Lenalidomide treatment, samples were stratified into “most sensitive” and “most resistant” groups based on the results of ex vivo DSRT. Patient samples with a low drug sensitivity score (DSS) are considered “most sensitive” to the individual drug being evaluated (Figure 3A,B).

### 3.5. Phosphoproteomic Analysis of CD138+ Myeloma Cells Based on Sensitivity/Resistance to Proteasome Inhibtors

**Figure 4:**
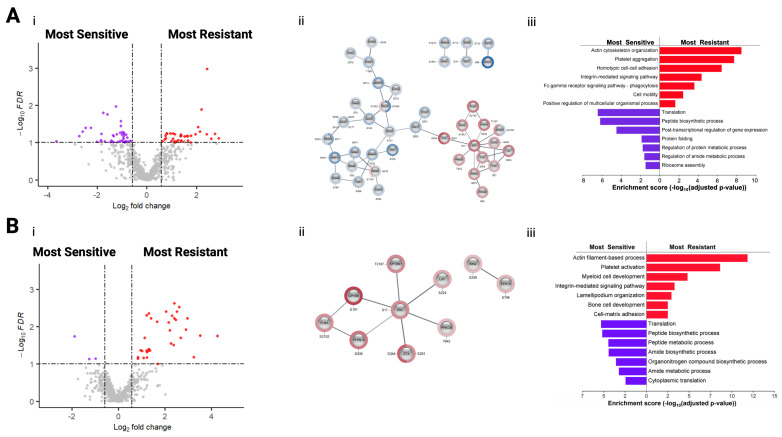
(**A**) Volcano plots of CD138+ myeloma cells considered “most sensitive” and “most resistant” to Bortezomib. (**i**) Volcano plot depicting SSDA phosphorylation sites. Purple points represent proteins/phosphosites increased in abundance in samples considered most sensitive to Bortezomib. Red points represent proteins/phosphosites considered most resistant to Bortezomib. (**ii**) Protein–protein interaction (PPI) network of phosphorylation sites upregulated in MM samples considered most sensitive (blue) and most resistant (red) to Bortezomib. (**iii**) g:Profiler analysis of proteins and phosphoproteins upregulated in myeloma cells most resistant (red bars) and most sensitive (purple bars) to Bortezomib. Go terms enriched biological processes highlighted as key terms in the g:Profiler analysis. (**B**) Volcano plots of CD138+ myeloma cells considered “most sensitive” and “most resistant” to Lenalidomide. (**i**) Volcano plot depicting SSDA phosphorylation sites. Purple points represent proteins/phosphosites increased in abundance in samples considered most sensitive to Lenalidomide. Red points represent proteins/phosphosites considered most resistant to Lenalidomide. (**ii**) Protein–protein interaction (PPI) network of phosphorylation sites upregulated in MM samples considered most resistant (red) to Lenalidomide. Phosphorylation sites associated with Lenalidomide sensitivity did not show connectivity. (**iii**) g:Profiler analysis of proteins and phosphoproteins upregulated in myeloma cells most resistant (red bars) and most sensitive (purple bars) to Lenalidomide. GO terms represent enriched biological processes highlighted as key terms in the g:Profiler analysis.

The phosphoproteomic profiles of MM patient samples considered most sensitive and most resistant to Bortezomib were compared in order to identify phosphorylation sites associated with response to these proteasome inhibitors (PIs). A total of 41 phosphorylation sites were increased in abundance in most resistant samples, and 50 phosphorylation sites were increased in abundance in most sensitive samples (FDR q-value < 0.1, FC > 1.5) (Figure 4(Ai)). To analyse the phosphoproteomic dataset, the Cytoscape app “Omics Visualizer” (Version 2001–2016) was used to visualise site-specific information on using STRING (version 12.0) analysis of functional protein association networks (Figure 4(Aii)). The minimum required interaction score was set to high confidence (0.7) and unconnected nodes were removed. Two clusters clearly separate the phosphoproteins associated with Bortezomib sensitivity, which are linked to RNA processing, and Bortezomib resistance, which include proteins connected to cytoskeleton and integrin-mediated signalling. GO analysis of the phosphoprotein results clearly determined an increase in adhesion and motility-linked biological processes in Bortezomib-resistant MM patient cells, whereas protein translation and protein folding-associated biological processes were increased in Bortezomib-sensitive MM patient cells (Figure 4(Aiii)). KSEA was utilised to predict potential kinase activity based on the phosphorylation levels of known substrates. This analysis highlighted a significant enrichment of eight kinases in Bortezomib-resistant samples and one kinase in Bortezomib-sensitive samples. The NF-κB signalling pathway-associated kinase IKBKB (Inhibitor of Nuclear Factor Kappa B Kinase Subunit Beta) and CHUK (Component Of Inhibitor Of Nuclear Factor Kappa B Kinase Complex), which make up the catalytic subunits of the multimeric I kappa B kinase (IKK) complex, displayed higher activity in Bortezomib samples, whereas the catalytic subunit of CK2 (Casein Kinase 2), CSNK2A1 (Casein Kinase 2 Alpha 1), showed higher activity in Bortezomib-sensitive samples. Lists of the top 10 phosphorylation sites associated with Bortezomib resistance and sensitivity are described in Table 2 and Table 3, respectively.

### 3.6. Phosphoproteomic Analysis of CD138+ Myeloma Cells Based on Sensitivity/Resistance to Immunomodulatory Drugs

The phosphoproteomic profiles of MM patient samples considered most sensitive and most resistant to Lenalidomide were compared in order to identify proteins and phosphorylation sites associated with response to this type of immunomodulatory drug (IMiDs). Volcano plot analysis identified 35 phosphorylation sites increased in abundance in the most resistant samples and 3 phosphorylation sites increased in abundance in the most sensitive samples (FDR q-value < 0.1, FC > 1.5; Figure 4(Bi)). STRING network analysis using “Omics Visualizer” identified a single cluster of phosphosites increased in abundance in Lenalidomide-resistant MM patient cells linked to actin filament organisation (Figure 4(Bii)). The minimum required interaction score was set to high confidence (0.7) and unconnected nodes were removed. KSEA did not detect any significant kinase enrichments. GO analysis of the phosphoprotein results demonstrated an increase in adhesion and motility-linked biological processes in Lenalidomide-resistant MM patient cells, while protein translation and metabolism-associated biological processes were increased in Lenalidomide-sensitive MM patient cells (Figure 4(Biii)). Subsequently, a list of the phosphorylation sites most significantly increased in Lenalidomide-resistant and Lenalidomide-sensitive samples was established (Table 4 and Table 5).

## 4. Discussion

Proteomics approaches are increasingly used to examine the biological basis of disease, identify new targetable proteins and pathways for therapeutic intervention, predict disease outcome/treatment response/side effect profiles, and uncover novel resistance mechanisms. Phosphoproteomics represents an essential “omics” approach to provide insight into the post-translational events involved in the pathogenesis of various conditions. As recent studies have demonstrated, the clinical applicability of ex vivo DSRT in investigating novel therapeutic targets and surrogate makers of drug sensitivity/resistance have significant clinical relevance [21,22]. In this study, proteins and phosphorylation sites were identified in MM patient cells that are associated with sensitivity/resistance to a selection of drugs based on ex vivo DSRT. This highlights the ability to combine high-sensitivity proteomics with ex vivo DSRT to investigate mechanisms of drug resistance, detect predictive markers of drug response and identify potential targets to circumvent drug resistance. MM patient cells are also considered more biologically representative of MM than panels of cell lines, therefore improving the clinical relevance of results derived from ex vivo DSRT in combination with proteomics.

Label-based mass spectrometry (LFQ) analysis identified a clear distinction in the phosphoproteomic profiles of MM patient cells in the very sensitive and very resistant chemosensitivity groups. Functional enrichment analysis uncovered an increased abundance of phosphoproteins associated with cell adhesion and cytoskeletal organisation in drug-resistant samples, confirming the strong link between cell adhesion molecules and drug resistance in MM [23,24]. The reduced levels of proteins involved in protein translation and ribosome functionality also indicate a slower cycling rate than drug-sensitive MM patient cells, a process that reduces the susceptibility of these cells to chemotherapies such as Bortezomib [25]. These results validate with known mechanisms of drug resistance, namely, Cell Adhesion-Mediated Drug Resistance (CAM-DR) and the persistence of quiescent cancer cells [26,27].

KSEA identified an enrichment of substrates for several kinases including PKA and protein kinase C beta (PRKCB). PKA is known to phosphorylate Filamin A, alpha (FLNA) at S2152 and increased activity may contribute to resistance mechanisms in MM [28]. Hyperphosphorylation at threonine 642 of PRKCB, a phosphorylation event that is essential for its enzymatic activity, was identified in Group 4 samples, suggesting an association between increased PRKCB activity and chemoresistance in MM [29]. There are indications that reduced PRKCB activity may enhance the chemosensitivity groups, highlighting proteins, phosphorylation sites, and predicted kinases that may play roles in general drug resistance mechanisms. Furthermore, phosphorylation sites commonly altered in abundance across both drugs investigated in this study can provide insight into the general mechanisms of resistance in MM patient cells.

Evaluating the phosphoproteomic profiles of MM patient cells grouped based on their ex vivo sensitivity to hundreds of drugs provides essential information on general or prominent resistance mechanisms, by examining the molecular profiles of MM cells considered resistant/sensitive to individual drugs. This approach can be used to identify resistance mechanisms and predictive biomarkers specific to the individual drugs being investigated. This phosphoproteomic analysis identified phosphorylation events on serine 65 (S65) and serine 82 (S82) of Heat shock protein 27 (Hsp27) that were increased in abundance in Bortezomib-resistant MM cells. Hps27 phosphorylation results in the formation of smaller oligomers with enhanced chaperone activity which prevents protein aggregation [30]. A recent study revealed a vital role of Hsp27 in the mechanism of action of Bortezomib, a therapeutic evaluated in this study. MM patients who responded to a Bortezomib-based treatment regimen had significantly lower expression of Hsp27 compared to those who did not respond to a Bortezomib-based treatment. Furthermore, treatment of MM cell lines with the Hsp27 inhibitor, Apatorsen (OGX-427), resulted in similar apoptosis rates and expression patterns of Hsp27, Bcl-2 (B-cell lymphoma 2) and Bax (Bcl-2-associated X protein) [31]. Blockade of Hsp27 has been reported to restore sensitivity in Bortezomib-resistant cells, while ectopic expression of Hsp27 rendered Bortezomib-sensitive cells resistant to Bortezomib treatment subsequently [32]. This underscores the importance of combining ex vivo DSRT with phosphoproteomics to identify key proteins and phosphorylation sites connected with drug resistance.

Filamin A is an actin-binding protein which acts as a scaffold for various protein partners, including transmembrane proteins such the integrins, a large family of heterodimeric (αβ) transmembrane receptors that mediate cell–matrix and cell–cell adhesion. Phosphorylation of FLNA at S2152 is well-documented, with several studies reporting that this phosphorylation event prevents cleavage of full-length (280 kDa) FLNA by calpains at the hinge region to the 110 kDa fragment that is further cleaved into a 90 kDa fragment [33,34]. Full-length FLNA is usually found in the cytoplasm, whereas the 90 kDA fragment is translocated into the nucleus, although some studies have reported the presence of full-length FLNA within nucleolus [35]. A study on prostate cancer reported differential pathological functions of cytoplasmic and nuclear FLNA, whereby cytoplasmic FLNA was linked to metastatic potential, while nuclear FLNA prevented cell invasion, highlighting the role of FLNA cleavage and thus S2512 phosphorylation in cancer [34]. Protein levels of FLNA were also increased in Bortezomib-resistant samples; however, the phosphorylation status of S2152 may provide more insight into the biological function and cellular location of FLNA in drug-resistant myeloma. Interestingly, nuclear FLNA supresses the transcription of ribosomal RNA by preventing the recruitment of RNA polymerase I to the rDNA promoter. The susceptibility of MM cells to proteasome inhibition is largely based on the high protein biosynthetic rate (to produce, fold, and secrete thousands of antibody molecules per second) of MM cells, which may result in dependence on the proteasome to remove misfolded proteins. Therefore, one of the resistance mechanisms of Bortezomib that has been proposed is the reduction in protein synthesis and proteasome workload [36,37]. Increased levels of FLNA may contribute to reducing the rate of protein synthesis in Bortezomib-resistant cells; however, further investigations are needed to support this hypothesis.

Interestingly, hypophosphorylation of serine 381 (S381) of TCOF1 was identified in Bortezomib-resistant MM cells, while protein levels of TCOF1 were unchanged across the two groups. TCOF1 encodes the treacle phosphoprotein, which is known to have roles in ribosome biogenesis and the DNA damage response [38,39]. Limited studies have investigated the effects of S381 on the function of TCOF1; however, this site has been found to be hyperphosphorylated in various cancers, including breast, colon, and ovarian [40]. KSEA revealed an enrichment of substrates of CK2 in Group 1 samples and those considered most sensitive to Bortezomib. CK2 has a range of diverse roles in carcinogenesis, and MM cells have been reported to rely on the activity of CK2 for survival [41]. CK2 stimulates the transcription of RNA polymerases, which are required for rRNA synthesis and subsequent protein translation to support cell growth and proliferation [42,43]. Increased cell proliferation via CK2-based regulation of signalling pathways such as NF-κB and PI3K/AKT/mTOR, and increased protein synthesis stimulated by CK2, could potentially increase the sensitivity of MM cells to various drugs due to the increased activity of their specific targets.

KSEA of Bortezomib-resistant MM showed an enrichment of substrates of IKBKB and CHUK kinases, which constitute the two catalytic subunits of the IKK complex that acts as a central regulator for the activation of NF-κB signalling [44]. Activation of the IKK complex results in the phosphorylation of IκB, leading to its proteasomal degradation, which releases NF-κB dimers, enabling their translocation to the nucleus where they stimulate the transcription of a myriad of genes involved in the inflammatory response, cell growth, and survival [45]. An important rationale for the use of PIs in the treatment of MM is to inhibit a key signalling pathway in MM pathogenesis, the NF-κB pathway. However, since the introduction of PIs, studies have found that Bortezomib also induces the activation of the canonical NF-κB pathway via the downregulation of IκB. Signalling via IKBKB has been found to play a key role in Bortezomib-induced NF-κB activation and the combination of Bortezomib with an IKBKB inhibitor has been reported to enhance Bortezomib-induced cytotoxicity [46,47]. Numerous studies have implicated NF-κB activity in Bortezomib resistance [48,49]. Increased activity of IKBKB and CHUK kinases may contribute to Bortezomib resistance and necessitate further investigation as targets in Bortezomib-resistant MM.

The activating phosphorylation site of tyrosine-protein kinase Fyn, S21, was determined to be hyperphosphorylated in Group 4 samples, representing the patient cohort considered most resistant to Bortezomib, while the phosphorylation of S558 on Fyn-binding protein was also found to be hyperphosphorylated in Group 4 samples. It has been reported in the literature that Fyn S21 is also a target of PKA [50]. Interestingly, CD45 has also been implicated in the activation of Fyn and other Src family kinases (SFKs) through the dephosphorylation of negative regulatory tyrosine phosphorylation sites. Hyperphosphorylation of CD45 and CD45-associated protein on serine 973 and serine 99, respectively, was identified in Group 4 samples compared to Group 1. Previous studies have reported that IL-6 signalling leads to the activation of SFKs, including Fyn in MM [51]. IL-6 has also been reported to induce CD45 expression, which is required for the activation of Fyn in MM cell lines [52,53,54,55]. Fyn activation has been linked to drug resistance in several cancers, including tamoxifen resistance in breast cancer and imatinib resistance in chronic myeloid leukaemia (CML) [56]. Another member of the SFKs, Src, demonstrated hyperphosphorylation on serine 17 in drug-resistant myeloma cells. Phosphorylation of Src at this site by PKA has been implicated in the activation of the small GTPase Rap1, which is involved in cell adhesion and extracellular signal-regulated kinases (ERKs) [57]. The role of Rap1 in regulating integrin activation has resulted in many investigations suggesting the targeting of Rap1 to combat CAM-DR [58,59,60]. Rap1 levels, which showed a greater than 4-fold increase in Group 4 samples compared to Group 1, has also been linked to chemotherapy response in breast cancer whereby increased Rap1 levels predict a poor response [61]. Therefore, PKA-mediated Src phosphorylation and the subsequent activation of Rap1 could potentially represent a mechanism of drug resistance in MM.

Of the phosphorylation sites found to be hypo- or hyperphosphorylated in this study, the effect that many of these phosphorylation events have on protein function is poorly understood. As an example, tyrosine-protein kinase BAZ1B phosphorylation at S1468 was discovered to be increased in Bortezomib-sensitive MM cells. An RNA interference screen identified the BAZ1B gene as a Bortezomib sensitizer in MM; however, the effect of S1468 phosphorylation on the function of BAZ1B is still to be uncovered [62]. Although phosphorylation events including dematin S16, large proline-rich protein BAG6 S1081, and kalirin S1799, are listed in the PhosphoSitePlus database, many have only been identified in large-scale mass spectrometry phosphoproteomic screens with limited information in relation to their functional and biological significance.

A limited number of clinical research groups have access to ex vivo drug screening platforms. Therefore, the integration of molecular profiling strategies such as phosphoproteomics to establish predictive biomarker panels that can act as surrogate markers of ex vivo drug sensitivity resistance testing is critical to expand the applicability of this precision medicine facilitator. To validate our findings, large-scale studies with sufficient statistical power are required to translate these preclinical findings into clinically relevant predictive assays. A large-scale comprehensive study combining extensive clinical data on outcomes, cytogenetics, DSRT and downstream proteomics technologies would undoubtedly aid in the translation on the results presented in this study to the clinical setting.

## 5. Conclusions

PTMs such as phosphorylation, acetylation, glycosylation, and ubiquitination are considered attractive therapeutic targets and/or predictive biomarkers because they act as molecular switches that regulate protein activity, stability, and localization, and are often dysregulated in diseases like cancer. Our study confirms that the combination of phosphoproteomics and ex vivo DSRT can provide novel insights into the biological mechanisms associated with drug resistance/sensitivity. An untargeted MS approach for identifying and quantifying phosphorylation sites provides a comprehensive, discovery-driven “shotgun” phosphoproteomics strategy that aims to map thousands of phosphorylation sites across the proteome without prior knowledge of which proteins are modified (as compared to targeted antibody-based analysis), as demonstrated in the investigation. Several well-documented mechanisms of resistance were identified in this study, reaffirming the confidence in the use of this approach to detect novel resistance mechanisms and predictive biomarkers of drug response. Results indicate an increase in cell adhesion-associated processes and a decrease in cell growth via decreased protein translation in multidrug-resistant MM cells based on ex vivo DSRT. Phosphorylation sites and kinases associated with drug resistance were identified and further studies should investigate the potential involvement of these kinases in the development of resistance to the individual drugs analysed in this study. Although information on the biological functions of many of the phosphorylation sites identified in this study are limited due to their novel nature, it is hoped that future studies evaluating the impact of these phosphorylation events on biological processes will provide insight into the potential links between these phosphorylation events and drug resistance.

## Figures and Tables

**Table 1:** Sample groupings based on drug sensitivity/resistance. Samples were grouped based on ex vivo DSRT results, as described in [12].

Group 1	Group 2	Group 3	Group 4
R_MM_6261	R_MM_1878	R_MM_3823	R_MM_1913
R_MM_7171	R_MM_6385	D_MM_7396	D_MM_7281
R_MM_3792	D_MM_7276	D_MM_7983	R_MM_1193
R_MM_2662	D_MM_7746	D_MM_8095	R_MM_587
R_MM_4263	R_MM_6211	D_MM_8597	R_MM_8291

**Table 2:** Top 10 phosphorylated peptides (phosphor-site) displaying a significantly increased abundance in MM samples considered most resistant to Bortezomib.

Protein Name	Gene Name	Biological Function	Phosphosite	FDR q-Value	FC
Heat shock protein beta-1	*HSPB1*	Molecular chaperone	S65	0	5.41
Proto-oncogene tyrosine-protein kinase Src	*SRC*	Cell adhesion	S17	0.013	4.6
Zyxin	*ZYX*	Cell adhesion	S281	0.038	4.19
Filamin-A	*FLNA*	Actin-binding	S2152	0.051	4.42
Transmembrane protein 40	*TMEM40*	Membrane protein	S137	0.057	1.75
Tyrosine-protein kinase Fyn	*FYN*	Adaptive immunity	S2152	0.057	2.08
Serine/arginine repetitive matrix protein 2	*SRRM2*	RNA binding	S1179	0.057	1.74
Zinc finger protein 609	*ZNF609*	Promoter-specific chromatin binding	S576	0.057	1.94
Zyxin	*ZYX*	Cell adhesion	S344	0.059	6.71
Sorting nexin-17	*SNX17*	Protein transport	S421	0.059	2.18

**Table 3:** Top 10 phosphorylated peptides (phosphor-site) displaying a significantly increased abundance in MM samples considered most sensitive to Bortezomib.

Protein Name	Gene Name	Biological Function	Phosphosite	FDR q-Value	FC
Treacle protein	*TCOF1*	Regulation of translation	S381	0.011	2.38
Coiled-coil domain-containing protein 86	*CCDC86*	RNA binding	S18	0.016	3.4
Major vault protein	*MVP*	Protein transport	S445	0.018	3.02
Lupus La protein	*SSB*	RNA binding	S366	0.027	2.07
DNA fragmentation factor subunit alpha	*DFFA*	Deoxyribonuclease inhibitor activity	S315	0.036	2.08
Serine/arginine-rich splicing factor 11	*SRSF11*	RNA binding	S449	0.041	4.76
Nuclease-sensitive element-binding protein 1	*YBX1*	Nucleic acid binding	S165	0.041	5.53
Tyrosine-protein kinase BAZ1B	*BAZ1B*	Transcription regulation	S1468	0.051	6.04
Suppressor of SWI4 1 homologue	*PPAN*	RNA binding	S359	0.052	1.98
Cyclin-dependent kinase 12	*CDK12*	Serine/threonine protein kinase	S423	0.054	2.11

**Table 4:** Top 10 phosphorylated peptides (phosphor-site) displaying a significantly increased abundance in MM samples considered most resistant to Lenalidomide.

Protein Name	Gene Name	Biological Function	Phosphosite	FDR q-Value	FC
Kalirin	*KALRN*	Guanine-nucleotide releasing factor	S1799	0.002	5.32
LIM domain and actin-binding protein 1	*LIMA*	Actin-binding	S490	0.003	6.15
Spectrin beta chain, non-erythrocytic 1	*SPTBN1*	Actin-binding	T2187	0.004	5.72
Protein FAM63A	*FAM63*	Cysteine-type deubiquitinase activity	S441	0.004	4.13
Briding integrator 2	*BIN2*	Cell chemotaxis	S259	0.004	2.48
Neurobeachin-like protein 2	*NBEAL2*	Protein kinase binding	S2739	0.005	2.32
Filamin-A	*FLNA*	Actin-binding	S2152	0.005	4.98
Serum deprivation-response protein	*SDPR*	Phosphatidylserine binding	S293	0.006	7.88
Zyxin	*ZYX*	Cell adhesion	S281	0.006	5.28
Epidermal growth factor receptor substrate	*EPS15*	Cadherin binding	S796	0.006	2.64

**Table 5:** Phosphorylated peptides (phosphor-site) displaying a significantly increased abundance in MM samples considered most sensitive to Lenalidomide.

Protein Name	Gene Name	Biological Function	Phosphosite	FDR q-Value	FC
ADP-ribosylation factor-like protein 6 interacting protein 4	*ARL6IP4*	RNA binding	S332	0.018	3.71
Cytoskeleton-associated protein 4	*CKAP4*	RNA binding	S26	0.073	2.39
Nuclease-sensitive element-binding protein 1	*YBX1*	Nucleic acid binding	S165	0.071	1.98

## Data Availability

The data presented in this study is available upon request from the corresponding authors. The data is not publicly available due to privacy and ethical constraints.

## Supplementary Materials

The following supporting information can be downloaded at: https://www.mdpi.com/article/10.3390/biom16020323/s1, Table S1: Clinical characteristics of patient cohort; Table S2: Details of treatment course of each patient within the cohort.

## Abbreviations

AGC	Automatic Gain Control
BAZ1B	Tyrosine-Protein Kinase BAZ1B
BM	Bone Marrow
CAM-DR	Cell Adhesion-Mediated Drug Resistance
CID	Collison-Induced Association
CK2	Casein Kinase 2
DSRT	Drug Sensitivity and Resistance
DSS	Drug Sensitivity Score
FLNA	Filamin A
GO	Gene Ontology
HCD	Higher Collison Dissociation
IKK	I Kappa B Kinase
IMiD	Immunomodulatory Drug
KSEA	Kinase-Substrate Enrichment Analysis
MM	Multiple Myeloma
PI	Proteasome Inhibitor
PKA	Protein Kinase A
PPI	Protein–Protein Interaction
PRKCB	Protein Kinase C Beta
SSDA	Statistically Significant Differentially Abundant
