# Supplementary material for: Phosphoproteomic Profiling of Multiple Myeloma Based on Ex Vivo Drug Sensitivity Resistance Testing Identifies Phosphorylation Signatures Associated with Drug Response
Source: Biomolecules. 2026 Feb 19;16(2):323. doi: 10.3390/biom16020323 (PMC12938258; doi:10.3390/biom16020323)
Supplement: Supplementary file 1 [file biomolecules-16-00323-s001.zip › biomolecules-4060858-supplementary.pdf]

Table S1 : Clinical characteristics of patient cohort. Table shows the patient ID, age at sample date, gender, ISS, heavy chain composition, and light chain composition of each sample.

Patient ID	Age at Sample Date	Gender	ISS	Heavy Chain	Light Chain
D_MM_7276	65	Female	2	Not detected	kappa
D_MM_7281	77	Female	2	IgA	kappa
D_MM_7396	50	Male	1	IgG	kappa
D_MM_7746	67	Female	3	IgG	lambda
D_MM_7983	63	Male	NA	IgG	lambda
D_MM_8095	77	Male	NA	IgA	kappa
D_MM_8597	53	Male	2	IgG	lambda
R_MM_1193	74	Male	3	IgA	lambda
R_MM_1878	69	Male	1	IgA	kappa
R_MM_1913	69	Female	2	IgG	kappa
R_MM_2662	62	Male	2	IgG	kappa
R_MM_3792	68	Male	3	IgG	kappa
R_MM_3823	71	Female	1	IgA	lambda
R_MM_4263	58	Female	NA	IgG	kappa
R_MM_587	72	Female	1	IgG	kappa
R_MM_6211	64	Female	2	Not detected	kappa
R_MM_6261	53	Male	3	IgG	kappa
R_MM_6385	76	Male	NA	IgA	lambda
R_MM_7171	70	Male	NA	Unknown	Unknown
R_MM_8291	71	Male	NA	Unknown	kappa

Table S2 : Details of treatment course of each patient within the cohort. Table showing the disease stage at time of sampling, response to treatment , the 1st next line of treatment, all lines of treatment, and the deepest response in next line of treatment. Bor, bortezomib; Dxm, dexamethasone; Cpm, cyclophosphamide; HD-Cyc, high-dose cyclophosphamide; AutoHSCT, autologous hematopoietic stem cell transplantation; HD-Mel, high-dose melphan; Mel, melphan; Pred, prednisone; Len, lenalidomide; Dara, daratumumab; VGPR, very good partial response; PR, partial response; PD, progressive disease; SD, stable disease.

Patient ID	Disease stage at sample date	Treatment Response at sample date	Name of 1st next line treatment	Names of all next line treatments	Deepest response in next line treatment
D_MM_7276	Diagnosis	Not treated	Bor/Dxm	Bor/Dxm Bor/Cpm/Dxm Mobilisation(HD-Cyc) Bor/Cpm/Dxm AutoHSCT (HD-Mel)	VGPR
D_MM_7281	Diagnosis	Not treated	Bor/Mel/Pred	Bor/Mel/Pred	VGPR
D_MM_7396	Diagnosis	Not treated	Bor/Cpm/Dxm	Bor/Cpm/Dxm Mobilisation (Cpm) AutoHSCT (HD-Mel) Bor/Dxm/Len Len	PR
D_MM_7746	Diagnosis	Not treated	Ixazomib/Len/Dxm	Ixazomib/Len/Dxm	PR
D_MM_7983	Diagnosis	Not treated	NA	NA	NA
D_MM_8095	Diagnosis	Not treated	Bor/Dxm	Bor/Dxm Radiation Therapy	VGPR
D_MM_8597	Diagnosis	Not treated	Ixazomib/Len/Dxm	Ixazomib/Len/Dxm AutoHDSCT (HD-Mel)	VGPR
R_MM_1193	Relapse	Triple Refractory	Carfilzomib/Dxm	Bor/Mel/Cpm/Doxorubicin	PD
R_MM_1878	Relapse	Single Refractory	Bor/Mel/Cpm	Carfilzomib/Elotuzumab/Dxm	VGPR
R_MM_1913	Relapse	Single Refractory	Bor/Mel/Cpm/Len	Carfilzomib/Elotuzumab/Dxm	PR
R_MM_2662	Relapse	Single Refractory	Bor/Len/Thalidomide	Carfilzomib/Elotuzumab/Dxm	VGPR
R_MM_3792	Relapse	Single Refractory	Bor/Mel/Cpm/Len	NA	NA
R_MM_3823	Relapse	Single Refractory	Bor/Mel/Cpm/Len	NA	NA
R_MM_4263	Relapse	Single Refractory	Bor/Mel/Cpm	Len/Dxm	NA
R_MM_587	Relapse	Single Refractory	Bor/Mel/Cpm/Thalidomide/Len	N/A	SD
R_MM_6211	Relapse	Single Refractory	Bor/Mel/Cpm	Bor/Dxm/Len	PD

R_MM_6261	Relapse	Single Refractory	Bor/Mel/Cpm/Len	Dara/Bor/Dxm	PD
R_MM_6385	Relapse	Triple Refractory	Bor/Mel/Cpm/Len	Treatment related to a study	VGPR
R_MM_7171	Relapse	Single Refractory	Bor/Mel/Cpm/Carfilzomib	NA	NA
R_MM_8291	Relapse	Single Refractory	Bor/Mel/Cpm	Carfilzomib/Elotuzumab/Dxm	VGPR
